# Prediction of beauty and liking ratings for abstract and representational paintings using subjective and objective measures

**DOI:** 10.1371/journal.pone.0200431

**Published:** 2018-07-06

**Authors:** David M. Sidhu, Katrina H. McDougall, Shaela T. Jalava, Glen E. Bodner

**Affiliations:** Department of Psychology, University of Calgary, Calgary, Alberta, Canada; Coventry University, UNITED KINGDOM

## Abstract

Recent research on aesthetics has challenged the adage that “beauty is in the eye of the beholder” by identifying several factors that predict ratings of beauty. However, this research has emerged in a piecemeal fashion. Most studies have examined only a few predictors of beauty, and measured either subjective or objective predictors, but not both. Whether the predictors of ratings of beauty versus liking differ has not been tested, nor has whether predictors differ for major distinctions in art, such as abstract vs. representational paintings. Finally, past studies have either relied on experimenter-generated stimuli—which likely yield pallid aesthetic experiences—or on a curation of high-quality art—thereby restricting the range of predictor scores. We report a study (N = 598) that measured 4 subjective and 11 objective predictors of both beauty ratings and liking ratings, for 240 abstract and 240 representational paintings that varied widely in beauty. A crossover pattern occurred in the ratings, such that for abstract paintings liking ratings were higher than beauty ratings, whereas for representational paintings beauty ratings were higher than liking ratings. Prediction was much better for our subjective than objective predictors, and much better for our representational than abstract paintings. For abstract paintings, liking ratings were much more predictable than beauty ratings. Implications and directions for future research are discussed.

## Introduction

A straw man in aesthetics research is the adage that “beauty is in the eye of the beholder”, ergo attempts to predict aesthetic judgments will be futile. Although this straw man often makes an appearance in articles on aesthetic judgments (we are guilty of this too), it is endorsed by few, if any, modern researchers. Indeed, Fechner’s [1] studies of aesthetic evaluations weakened this straw man long ago, and in doing so gave both weight and strength to the idea that subjective, evaluative judgments are lawful and hence predictable [1].

In our view, the question of whether aesthetic judgments are predictable should be updated with a refined set of questions. Is prediction better based on subjective ratings of stimuli, or based on objectively quantifiable properties of the stimuli? Is the ability to predict aesthetics judgments better for some classes of stimuli than others (e.g., abstract vs. representational paintings)? And how is the constellation of relevant predictors influenced by the type of aesthetic judgment participants are asked to make (e.g., beauty vs. liking)? Our study attempts to answer this refined set of questions, and in doing so, it helps position aesthetics research for posing and wrestling with a fascinating set of “why” questions that follow from our findings. Why is the prediction of aesthetic judgments better for subjective ratings than for objective measures? Why is prediction better for representational paintings than for abstract paintings? And why are liking ratings sometimes more predictable than beauty ratings? Answering both sets of questions will inform and constrain our understanding of the basis of aesthetic judgments—a rapidly emerging area of research and theorizing (for reviews, see [2–4]).

### Prediction for subjective vs. objective measures

Research focused on predicting aesthetic responses to artworks has generally relied on the collection of either subjective [5, 6] or objective measures [7–9]. Subjective measures involve asking participants to rate a set of stimuli on a given dimension thought to influence aesthetic judgments. The most extensively normed set of subjective ratings of paintings collected to date, the JenAesthetics database, was provided by Amirshahi et al. [5]. In their study, participant ratings included how much they liked the color, content, and composition of a large set of images of paintings that spanned many content domains (e.g., abstract, landscape, still life, portrait, nude, urban scene). Participants provided each of these ratings for a subset of 163 paintings from a larger set, and they also rated both the beauty and “aesthetic quality” of these same paintings. Analysis primarily focused on the relationship among the subjective ratings (all of which were positively correlated). Since the influential line of work of Berlyne [10], a wide range of subjective dimensions of artworks have been argued to be predictive of aesthetic experiences, including meaningfulness, emotionality, complexity, color warmth, familiarity/novelty, interestingness, and prototypicality (for a review, see [3]). Although objective measures currently exist for some of these dimensions, such as complexity [8]), they have typically been gauged using participants’ subjective ratings.

The objective approach to predicting aesthetic experiences, pioneered by Fechner [1], involves measuring the “statistical image” properties of paintings via computational analysis. A wide range of objective measures have been investigated this way, including symmetry, self-similarity, complexity, aspects of composition (e.g., aspect ratio, deviation from the rule of thirds), spatial frequency power spectra, and color properties (e.g., hue, saturation, brightness; for a review, see [11, 12]). Objective measures have also been used to characterize the aesthetic properties of photographs [13], as well as to predict people’s preference for natural scenes over urban scenes [14, 15].

Hayn-Leichsenring, Lehmann, and Redies [12] examined how well a set of objective measures were able to predict both beauty and aesthetic ratings, using paintings from the Amirshahi et al. [5] norms. Multiple linear regression analyses revealed three significant objective predictors of beauty ratings (aspect ratio, color value, self-similarity) and these same three measures, plus an objective measure of complexity, were significant predictors of aesthetic ratings. The amount of variance explained by the objective predictors was not reported in these studies. Moreover, although correlations between subjective and objective measures were reported, the subjective predictors were not included in the regressions. Therefore, the opportunity to examine whether subjective or objective measures account for more variance in aesthetic ratings was missed. Similarly, Lyssenko, Redies, and Hayn-Leichsenring [16] examined how well the same set of objective predictors predicted a host of subjective ratings for a set of abstract artworks, but the subjective ratings were treated as outcome variables rather than as potential predictors of aesthetic ratings. Thus, to date no study has examined and compared the ability of both subjective and objective measures for predicting beauty ratings. Our study closed this gap.

Our methodology differed in another notable way from previous studies that have collected multiple subjective ratings [5, 6, 16]. In prior studies, participants rated each painting on all of the subjective dimensions, with the ratings collected in a constant order. This within-subject approach risks carryover effects. For example, if a given painting is rated low on one subjective dimension, participants may also tend to rate it low on the other subjective dimensions they are rating. This could result in an attenuation of differences among the subjective predictors. To eliminate this risk, in the present study separate sets of participants provided each predictor or outcome rating.

### Prediction for abstract vs. representational paintings

A major distinction in painted artworks is between abstract and representational styles. In contrast to representational paintings (landscapes, still lives, portraits, etc.), abstract paintings do not portray or evoke obvious, unambiguous semantic content. Past studies of aesthetic ratings have typically either used only abstract artworks [7, 17] or only representational artworks [6], or else they collapsed across a variety of painting types in analysis ([5, 12]; but see [11]). However, in an influential study, Vessel and Rubin [18] reported greater agreement across individuals regarding the beauty of representational images relative to abstract images (the stimuli were not artworks). They argued that the availability of semantic content for the representational images lead to the development of shared preferences across participants. Importantly, as they note, the finding that shared taste influences the experience of beauty rules out approaches to aesthetics that refer only to stimulus attributes. Given their findings, we included both subjective measures (which can be influenced by semantics and/or shared taste) and objective measures, and we included both abstract and representational paintings.

### Prediction for beauty vs. liking ratings

Research in aesthetics has focused on identifying the key factors that influence the perception of beauty in particular, typically as assessed by beauty ratings. Recently, however, Lyssenko et al. [16] have advocated for contrasting beauty judgments with other aesthetic judgments such as aesthetic quality [6, 16]. These researchers have begun to compare the predictors of ratings of “beauty” to the predictors of ratings of aesthetic quality [12, 16]. The claim is that beauty ratings capture subjective liking of the stimulus, whereas aesthetic ratings are intended to capture the “more objective” artistic value of the stimulus. Although this research has found somewhat different predictors for the two ratings, these studies do not report the correlation between them. Moreover, we are unsure how subjective judgments of “aesthetics” made by participants could be construed either by the participants or by the researchers as objective. We assume the distinction being captured here is between the paintings the participants themselves like (beauty ratings) and the paintings the participants believe others will generally like (aesthetic ratings) and thus both are subjective measures.

We heeded Lyssenko et al.’s [16] advice to collect more than one aesthetic rating, but rather than asking participants to attempt to make “objective” aesthetic quality judgments, we simply asked some of them to make liking ratings [6, 16]. We then examined, for the first time, whether subjective and/or objective predictors of beauty contrast with predictors of liking, for abstract and/or representational paintings. We suggest that liking ratings provide a potentially useful contrast to beauty ratings given that some people may like artworks that they do not experience as beautiful (e.g., “bad art”, see [9]).

### Prediction for real paintings wide-ranging in quality

The experimental approach to studying aesthetic responses has often relied on the creation of stimuli, such as dot patterns or geometric patterns, that vary on dimensions such as symmetry and/or complexity, and to then compare aesthetic ratings as a function of those dimensions (see [19, 20]). Using this approach, Tinio and Leder [20] found that symmetry was a stronger predictor of beauty ratings than was complexity, for example. The ability to control the dimensionality of one’s stimuli can be an asset. However, a trade-off to greater experimental control is the risk that these experimenter-created stimuli yield only pallid reactions (often around the midpoint of the scale) rather than genuine experiences of beauty. Participants may dutifully place the stimuli between rating scale anchors of “least” vs. “most” beautiful in a relative sense, while not finding any of them beautiful in an absolute sense. To risk overstating the point, it is unlikely that the raters would, for example, wish to hang a print on their wall of even the most symmetrical and complex geometric pattern from these studies. Because we wished to study the predictors of the aesthetic reactions of “beauty” and “liking” we therefore used images of real painted artworks. This also served to increase the generalizability of our results.

In addition, we intentionally selected images of paintings that spanned a wide range of beauty, as identified in a previous study [21]. Were we to have used only high beauty “gallery quality” paintings we would have exposed ourselves to two pitfalls: (1) restricted range on our outcome variables and on at least some of our predictor dimensions, and (2) an increased likelihood that some participants would be familiar with some of the paintings, which could well influence their aesthetic ratings. Indeed, Hayn-Leichsenring et al. [12] noted that a limitation of the Amirshahi et al.’s [5] JenAesthetics database is that it includes only high quality paintings. As a result, they noted that “any differences in aesthetic ratings of these images may be relatively small, and therefore the aesthetic ratings may be rather stable across art styles and subject matter” (p. 18). By selecting large sets of actual abstract and representational artworks that each varied widely in quality we therefore gave ourselves the best opportunity to detect differences in prediction as a function of measure type, painting type, and rating type.

## Materials and methods

### Ethics statement

This research was approved by the Conjoint Faculties Research Ethics Board at the University of Calgary. Participants gave informed consent via mouse click, received course credit for participating, and were debriefed after the study.

### Participants

University of Calgary undergraduates (N = 598; 449 female; *M* Age = 20.4, *SD* = 3.42) participated in an online study. About half rated abstract paintings while the other half rated representational paintings. Subsets of at least 40 participants rated their assigned painting type on one of the following dimensions: beauty, liking, meaningfulness, complexity, emotionality, or color warmth. Art expertise could not be examined because only 32 participants (5%) across the 12 groups who provided subjective measures self-identified as art experts, and their expertise was not independently assessed.

### Materials

The stimuli were 240 abstract and 240 representational paintings selected from online image databases (e.g., Artstor, Oxford Art Online) and Google searches that spanned a wide range of quality (see S1 Appendix for links to examples), as verified in recent work based on these stimuli [21]. The abstract paintings did not contain salient semantic or representational content. Most of the representational paintings were landscape scenes. Most of the paintings were not well-known or by famous artists, but a few paintings by somewhat well-known artists (e.g., Georgia O’Keefe) were included to achieve a wide range of quality. The images of the paintings were re-sized to 500 pixels on their longer dimension.

### Procedure

#### Dependent variables and subjective predictors

Each participant rated the 240 abstract or representational paintings on a single dimension. They were asked to rate paintings one at a time on a 9-point scale based on their automatic and spontaneous feelings for each painting. Two rated dimensions served as dependent variables: **beauty** (1 = ugly, 5 = neither ugly nor beautiful, 9 = beautiful) and **liking** (1 = dislike, 5 = neither dislike nor like, 9 = like). Based on prior research on aesthetics, we chose the following four subjective predictors: **meaningfulness** (1 = meaningless, 5 = neither meaningless nor meaningful, 9 = meaningful) [22], **complexity** (1 = simple, 5 = neither simple nor complex, 9 = complex) [19, 20, 23, 24], **emotionality** (1 = not emotional at all, 5 = neither not emotional nor very emotional, 9 = very emotional) [6, 25], and **color warmth** (1 = very cold in color, 5 = neither cold nor warm, 9 = very warm in color) [7, 26]. Participants were asked to use the entire range of the scale.

Participants were asked to complete the ratings in one session (of about 30 minutes), to set their browser to full screen mode so they could see each painting in its entirety without needing to scroll, and to avoid distractions (e.g., phone, email). Participants then viewed and rated their paintings presented in a randomized order. We computed a mean score for each painting, on each rated dimension.

#### Objective predictors

In addition to collecting subjective ratings, following Berman et al. [14] we used MATLAB’s Image Processing Toolbox [27] to quantify 11 perceptual/statistical properties of the images. These properties served as our objective predictors. We quantified the paintings’ color according to the hue, saturation, and value (henceforth brightness; HSV) model of color. **Hue** refers to the dominant wavelength of light from the color spectrum. **Saturation** refers to the intensity or “colorfulness” of a given color. **Brightness** refers to the brightness of a given color. A value on each of these dimensions was calculated for each pixel in an image, from which means and standard deviations (SD) were generated.

Mean hue makes a poor linear predictor because it is a cyclical dimension with arbitrary end points (i.e., moving beyond the highest value of hue yields the lowest value of hue). Therefore, we used the RGB model of color (i.e., the amount of red, green or blue light present; each of which functions well as a linear predictor) to quantify the hue of each painting using Adobe Photoshop. Preliminary analyses indicated high collinearity among red, green and blue luminance (after adjusting each by overall luminance), so a principal components analysis was used to reduce them to a single dimension that explained 74.20% of the variance. High values on this **RGB component** predictor correspond to low red luminance and high green and blue luminance.

**Entropy** refers to the unpredictability–or disorder–of the pixels in a painting. It was quantified by examining the frequency distribution of intensity values for all pixels in a given painting, after converting it to greyscale. More uniform distributions resulted in greater entropy values. Given the potential role of line orientation in aesthetic reactions [28, 29], we quantified the **straight edge density** and **non-straight edge density** (i.e., curved or fragmented edges) of each painting using a modification of Berman et al.’s [14] code. Finally, because several studies have suggested an important role for symmetry in aesthetic reactions [10, 19, 20, 30], we also quantified the **vertical symmetry** and **horizontal symmetry** of each painting by calculating the similarity between the first half of each image and the mirror image of its second half. Each half was converted into a vector of RGB intensity values, and the cosine similarity between these vectors was then taken. See S2 Appendix for more detail on these calculations. The data can be found in the following OSF repository: https://osf.io/2sy4f.

## Results

### Ratings

**Fig 1:**
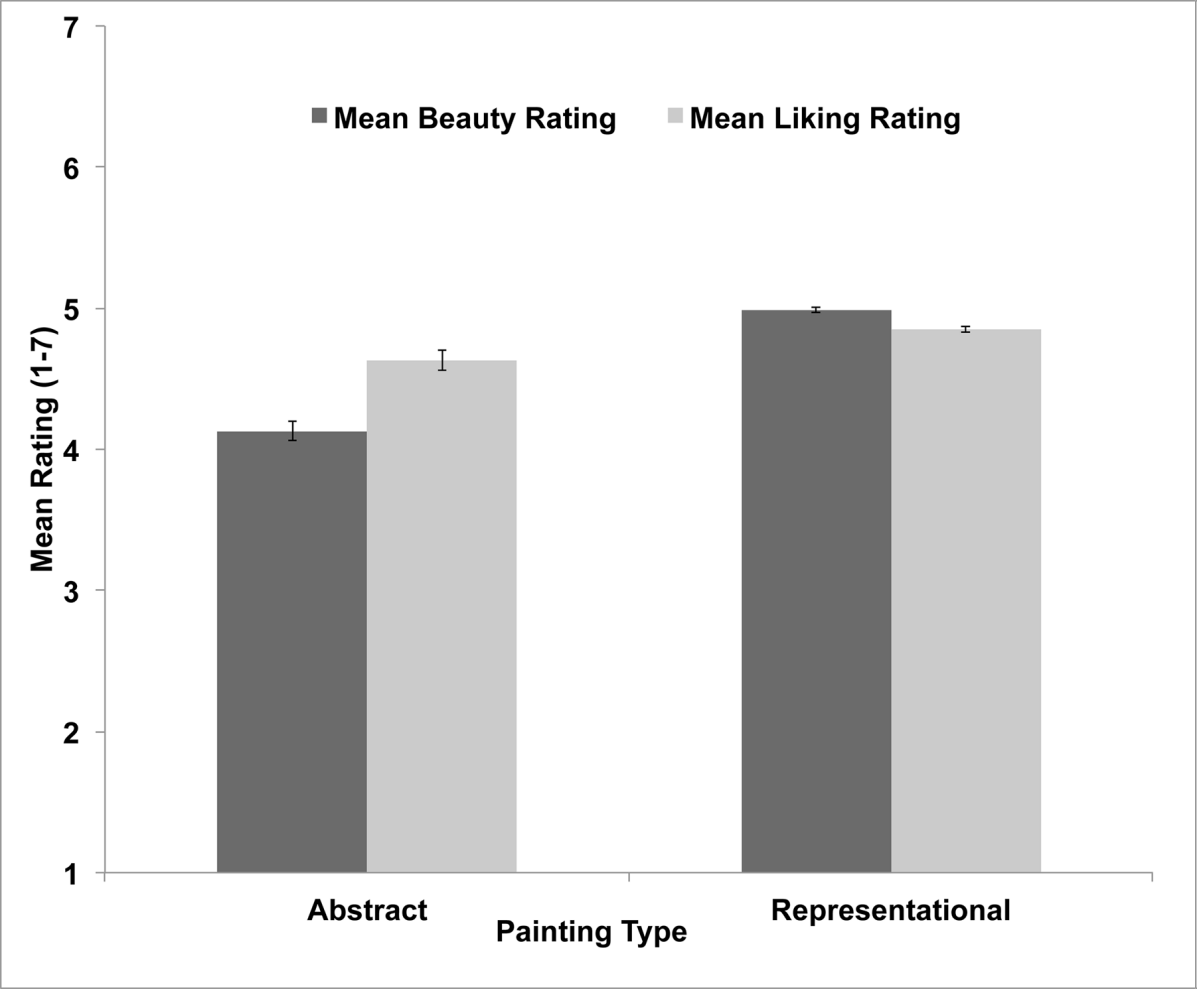
Mean beauty and liking ratings for abstract and representational paintings. Error bars represent 95% confidence intervals for which within-subjects variance has been removed using the approach described by Cousineau [31].

Our study was the first to examine both beauty and liking ratings for both representational and abstract paintings. Therefore, we first examined participants’ mean ratings in a 2 (painting type: representational vs. abstract) x 2 (rating type: beauty vs. liking) between-subjects ANOVA (see Fig 1). Overall, representation paintings yielded higher aesthetic ratings (*M* = 4.91, *SD* = 0.91) than abstract paintings (*M* = 4.38, *SD* = 0.71), *F*(1, 472) = 50.75, *MSE* = 1.33, *p* < .001. Liking ratings (*M* = 4.74, *SD* = 0.87) were also found to be higher than beauty ratings (*M* = 4.56, *SD* = 1.04), *F*(1, 472) = 22.90, *MSE* = 0.33, *p* < .001. As shown in Fig 1, the ANOVA also yielded a robust crossover interaction, *F*(1, 472) = 72.14, *MSE* = 0.33, *p* < .001. For abstract paintings, liking ratings (*M* = 4.62, *SD* = 0.77) were higher than beauty ratings (*M* = 4.14, *SD* = 1.00), *t*(235) = 6.95, *SE* = 0.07, *p* < .001, whereas for representational paintings, beauty ratings (*M* = 4.99, *SD* = 0.90) were higher than liking ratings (*M* = 4.85, *SD* = 0.95), *t*(237) = 6.03, *SE* = 0.02, *p* < .001. Thus, our art novice participants generally preferred representational over abstract paintings [18], and our analysis revealed that beauty and liking ratings diverge oppositely for abstract vs. representational paintings. Beauty ratings for abstract paintings were particularly low, a point to which we return below.

### Regression models

Respectively, Tables 1 and 2 provide the correlations among the 2 dependent measures and 4 subjective ratings for abstract and representational paintings. S1 Table and S2 Table provide the full correlation matrices (i.e., including the objective measures) for abstract and representational paintings, respectively. Many of the predictors were strongly correlated (particularly the subjective ratings for representational paintings), resulting in modest squared semi-partial correlations. However, the resulting models had VIF values below 10, indicating that multicollinearity was not a problem [32]; one exception, not based on VIF values, is mentioned below.

**Table 1:** Abstract paintings: Correlations between subjective ratings.

Subjective rating	1	2	3	4	5
(1) Beauty	–				
(2) Liking	.27***	–			
(3) Meaningfulness	.14*	.54***	–		
(4) Complexity	.30***	.32***	.55***	–	
(5) Emotionality	.35***	.62***	.68***	.59***	–
(6) Color Warmth	.04	–.17**	.01	.13	–.13*

* p < .05.

** *p* < .01.

*** *p* < .001.

**Table 2:** Representational paintings: Correlations between subjective ratings.

Subjective	1	2	3	4	5
(1) Beauty	–				
(2) Liking	.93***	–			
(3) Meaningfulness	.82***	.79***	–		
(4) Complexity	.79***	.71***	.79***	–	
(5) Emotionality	.77***	.73***	.88***	.75***	–
(6) Color Warmth	.33***	.37***	.18**	.24***	.11

* p < .05.

** *p* < .01.

*** *p* < .001.

For our main analyses, we report 4 multiple regressions that in turn examined the factors that predict either beauty or liking for either abstract or representational paintings. Each model was built using a bidirectional stepwise approach that was evaluated using the Bayesian Information Criterion (BIC) measure. BIC provides an estimate of the amount of information lost when a model is used to estimate a set of values. It penalizes models having more predictors, and thus it strives towards a balance between parsimony and goodness of fit. Beginning with no predictors in the model, the selection procedure either adds or drops the predictor that would lead to the largest decrease in BIC. This continues until adding or dropping a predictor would not improve BIC. S1 Appendix provides links to example paintings that were low or high on each significant predictor dimension. Model selection was conducted using R [33] and the MASS package [34]. The 4 subjective predictors were: meaningfulness, complexity, emotionality, and color warmth. The 11 objective predictors were: RGB color component, hue SD, saturation, saturation SD, brightness, brightness SD, entropy, straight edge density, non-straight edge density, vertical symmetry, and horizontal symmetry. In addition, for each type of painting, we report 2 exploratory multiple regressions that included either all 4 subjective predictors, or all 11 objective predictors. These regressions assessed the amount of variance captured by each type of predictor for each type of painting. Cohen’s *f*^2^ is also reported for each model as a measure of effect size. Although automated stepwise regression models tend to overfit data [35], S2 Appendix shows that similar results were obtained using a least absolute shrinkage and selection (LASSO) method [36].

#### Predicting beauty ratings for abstract paintings

In the model predicting beauty ratings for abstract paintings, meaningfulness was removed as a predictor because its coefficient changed from positive (as in its zero-order correlation with beauty rating) to negative, indicative of multicollinearity. Abstract paintings received higher beauty ratings when they were higher in emotionality (subjective) and entropy (objective). The remaining predictors were not significant. The Adjusted R^2^ was 0.13, Cohen’s *f*^2^ = 0.15 (Table 3).

**Table 3:** Regression model predicting beauty ratings for abstract paintings.

Variable	*B*	*SEB*	*β*	*sr*^*2*^	*VIF*
Subjective Predictors					
Emotionality	0.40	0.09	0.29	0.08***	1.11
Objective Predictors					
Entropy	0.28	0.11	0.17	0.03*	1.11

*B* = unstandardized coefficient; *SEB* = standard error of the computed coefficient; *β* = standardized coefficient; *sr*^2^ = squared semi-partial correlation; *VIF* = variance inflation factor; Adjusted R^2^ = 0.13; BIC = 654.58.

* *p* < .05.

*** *p* < .001.

The subjective-predictor model (i.e., all subjective predictors entered together) had an Adjusted R^2^ of 0.12, and the objective-predictor model (i.e., all objective predictors entered together) had an Adjusted R^2^ of only 0.04 (Table 4). Thus, the beauty of abstract paintings was not well captured by either our subjective or objective measures.

**Table 4:** Adjusted R^2^ values (Cohen’s *f*^2^ effect size) for the subjective and objective predictor regression models.

Rating Type/Painting Type	Predictor Type	
	Subjective	Objective
Beauty/Abstract	0.12 (0.14)	0.04 (0.04)
Liking/Abstract	0.42 (0.72)	0.24 (0.32)
Beauty/Representational	0.74 (2.85)	0.25 (0.33)
Liking/Representational	0.69 (2.23)	0.30 (0.43)

#### Predicting liking ratings for abstract paintings

Several more of our measures were predictive of liking ratings for abstract paintings than were predictive of beauty ratings, resulting in an Adjusted R^2^ of 0.56, Cohen’s *f*^2^ = 1.27 (Table 5). Indeed, the correlation between beauty and liking ratings for abstract paintings, though significant, was modest at *r* = .27, *p* < .001. Abstract paintings were liked more if they were higher in meaningfulness and emotionality (subjective), and had higher mean brightness, hue standard deviation, saturation standard deviation, and RGB component scores (objective). Conversely, abstract paintings were liked less if they had higher brightness SD (objective).

**Table 5:** Regression model predicting liking ratings for abstract paintings.

Variable	*B*	*SEB*	*β*	*sr*^*2*^	*VIF*
Subjective Predictors					
Meaningfulness	0.20	0.06	0.20	0.02**	1.88
Emotionality	0.52	0.06	0.49	0.12***	1.94
Objective Predictors					
Brightness Mean	1.02	0.21	0.22	0.04***	1.08
Hue SD	0.17	0.07	0.11	0.01*	1.08
Brightness SD	-1.61	0.51	-0.15	0.02**	1.21
Saturation SD	1.17	0.45	0.13	0.01*	1.23
RGB Component	0.17	0.03	0.27	0.07***	1.07

*B* = unstandardized coefficient; *SEB* = standard error of the computed coefficient; *β* = standardized coefficient; *sr*^2^ = squared semi-partial correlation; *VIF* = variance inflation factor

Adjusted R^2^ = 0.56; BIC = 396.00.

* *p* < .05.

** *p* < .01.

*** *p* < .001.

The subjective-predictor model had an Adjusted R^2^ of 0.42, whereas the objective-predictor model had an Adjusted R^2^ of only 0.24 (Table 4). Thus, the liking of abstract paintings was better captured by our subjective (vs. objective) measures, but allowing both types of measures to compete in stepwise regression yielded a model with the highest Adjusted R^2^.

#### Predicting beauty ratings for representational paintings

In general, prediction was much better for representational paintings than for abstract paintings. For beauty ratings, the overall Adjusted R^2^ was 0.81, Cohen’s *f*^2^ = 4.26 (Table 6). Representational paintings were deemed more beautiful if they were higher in meaningfulness, complexity, emotionality, and color warmth (subjective); and if they had higher brightness SD, horizontal symmetry, and RGB component scores (objective).

**Table 6:** Regression model predicting beauty ratings for representational paintings.

Variable	*B*	*SEB*	*β*	*sr*^*2*^	*VIF*
Subjective Predictors					
Meaningfulness	0.33	0.08	0.28	0.01***	5.53
Complexity	0.28	0.05	0.32	0.03***	3.34
Emotionality	0.31	0.08	0.24	0.01***	4.69
Color Warmth	0.18	0.03	0.25	0.03***	1.87
Objective Predictors					
Brightness SD	2.70	0.60	0.16	0.02***	1.64
RGB Component	0.26	0.04	0.21	0.03***	1.60
Horizontal Symmetry	1.48	0.38	0.14	0.01***	1.52

*B* = unstandardized coefficient; *SEB* = standard error of the computed coefficient; *β* = standardized coefficient; *sr*^2^ = squared semi-partial correlation; *VIF* = variance inflation factor

Adjusted R^2^ = 0.81; BIC = 274.36.

*** *p* < .001.

The subjective-predictor model had an Adjusted R^2^ of 0.75, whereas the objective-predictor model had an Adjusted R^2^ of only 0.25 (Table 4). Thus, liking of abstract paintings was better captured by our subjective (vs. objective) measures.

#### Predicting liking ratings for representational paintings

Unlike for abstract paintings, the pattern of predictors for representational paintings was very similar for beauty and liking ratings. This is not surprising given that beauty and liking ratings were very strongly correlated for representational paintings, *r* = .93, *p* < .001. Representational paintings received higher liking ratings if they were higher in meaningfulness, complexity, emotionality, and color warmth (subjective); and when they had higher brightness SD, horizontal symmetry, and RGB component scores (objective). Liking ratings were lower for representational paintings with higher straight edge density and non-straight edge density (objective). The model had an Adjusted R^2^ of 0.80, Cohen’s *f*^2^ = 4.00 (Table 7).

**Table 7:** Regression model predicting liking ratings for representational paintings.

Variable	*B*	*SEB*	*β*	*sr*^*2*^	*VIF*
Subjective Predictors					
Meaningfulness	0.37	0.09	0.29	0.01***	5.86
Complexity	0.22	0.05	0.24	0.02***	3.87
Emotionality	0.28	0.09	0.21	0.01**	5.24
Color Warmth	0.26	0.03	0.34	0.06***	1.90
Objective Predictors					
Brightness SD	3.02	0.66	0.17	0.02***	1.69
RGB Component	0.33	0.05	0.25	0.04***	1.61
Straight Edge Density	-7.41	2.62	-0.10	0.01**	1.50
Non-Straight Edge Density	-7.23	1.24	-0.20	0.03***	1.38
Horizontal Symmetry	1.94	0.42	0.17	0.02***	1.61

*B* = unstandardized coefficient; *SEB* = standard error of the computed coefficient; *β* = standardized coefficient; *sr*^2^ = squared semi-partial correlation; *VIF* = variance inflation factor

Adjusted R^2^ = 0.80, BIC = 318.32.

** *p* < .01.

*** *p* < .001.

The subjective-predictor model had an Adjusted R^2^ of 0.69, and the objective-predictor model had an Adjusted R^2^ of only 0.30 (Table 4), once again showing better prediction from our subjective (vs. objective) measures. Table 8 provides a summary of the significant predictors in each model.

**Table 8:** Summary of significant predictors for each regression model.

	Abstract Paintings		Representational Paintings	
Predictor	Beauty	Liking	Beauty	Liking
Subjective				
Meaningfulness		+	+	+
Complexity			+	+
Emotionality	+	+	+	+
Color Warmth			+	+
Objective				
Hue SD		+		
Saturation SD		+		
Brightness Mean		+		
Brightness SD		–	+	+
RGB Component		+	+	+
Entropy	+			
Straight Edge Density				–
Non-Straight Edge Density				–
Horizontal Symmetry			+	+

Sign indicates whether the predictor was positively or negatively related to a given outcome variable.

## Discussion

We examined how well subjective ratings and objective stimulus dimensions predict ratings of beauty and liking for abstract and representational paintings. Different sets of participants provided each subjective rating to avoid the potential for carry-over effects where a given rating is influenced by other ratings made by the same participant. Prior studies have explored aesthetic evaluations of either abstract [7, 17] or representational [6] artworks, or largely collapsed across various types of artwork in their data analysis ([5, 12]; but see [11]). By collecting independent sets of ratings for these two major classes of painting we were able to examine how two common types of positive aesthetic experience—beauty and liking—are influenced by this classic distinction in art.

Our study yielded several novel and intriguing findings. One was that our set of subjective measures accounted for 2–3 times more variance in ratings than did our set of objective measures (see Table 2). Thus, to reanimate the straw man from our introduction, it may be the case that beauty (and liking) are largely in the eyes of beholders—but—there is a good deal of consistency across beholders (at least when the beholders are art novices). This difference could reflect a fundamental distinction in the potency of subjective versus objective measures. Alternatively, it could reflect our particular arrays of measures. We attempted to capture many perceptual/statistical dimensions of the paintings, by extending the objective measures that Berman et al. [14] successfully used to identify some of the stimulus properties that account for human preference for natural vs. urban scenes. However, other sets of objective measures exist and could be explored in future studies [12, 16]. We opted to adapt the former set because they accounted for 31% of the variance in aesthetic preferences of images [15], whereas the predictive power of the latter set was not reported. Regardless, the inclusion of other objective predictors could potentially yield higher prediction rates, and this remains an important research direction.

Another intriguing finding was that prediction of beauty and liking ratings, respectively, was very respectable and far better for representational paintings (R^2^ = .81 and .80) than for abstract paintings (R^2^ = .13 and .56). This pattern fits with Vessel and Rubin’s [18] finding that taste for representational paintings is shared, relative to taste for abstract paintings which tends to be more idiosyncratic. Despite being correlated, each of our subjective predictors (meaningfulness, complexity, emotionality, color warmth) explained incremental variance in both beauty and liking ratings for at least one painting type, as did several objective predictors.

An interesting question for future research is whether a sole higher-order subjective dimension such as “quality” or “realness” or “familiarity” might underlie perceptions of beauty/liking, at least for representational paintings. On this issue, participants might adopt a consistent criterion for rating representational art but may be more variable in the criteria they adopt for rating abstract art. For example, raters might favor complex abstract art at first, but later shift to preferring simpler abstract artworks. This possibility would fit well with Vessel and Rubin’s [18] finding of greater variance in preference for abstract vs. representational images. The criteria used for evaluating paintings may be easier to verbalize for representational art (e.g., “I like the ones that look realistic”) than for abstract art (e.g., “I just know what I like”), and this may contribute to the greater predictability for representational paintings. It might also explain why ratings of the beauty of abstract paintings have been shown to be highly sensitive to context [7, 17]. Consistent with these possibilities, the standard deviation of beauty ratings was higher for abstract paintings (*M* = 2.08; *SD* = 0.24) than for representational paintings (*M* = 2.04; *SD* = 0.18), *t*(472) = 2.22, *SE* = 0.02, *p* = .03. Likewise, the standard deviation of liking ratings was higher for abstract paintings (*M* = 2.34; *SD* = 0.20) than for representational paintings (*M* = 2.25; *SD* = 0.19), *t*(472) = 4.72, *SE* = 0.02, *p* < .001. Alternatively, our effects of painting type could be due to stimulus-selection artifacts. For instance, our representational paintings were largely landscapes. Whether the same predictors explain variance in beauty/liking for other types of representational paintings (e.g., still life, portrait) remains to be seen.

Our decision to collect ratings of both beauty and liking also proved to be important. Collection of liking ratings for artworks [6, 16] allows for the possibility that observers sometimes like art that is not beautiful in a normative sense (and vice versa). For representational paintings, prediction was similar for the two types of ratings. For abstract paintings, in contrast, liking ratings were generally higher than beauty ratings, and prediction of liking ratings was much better. One take on the latter outcome is that art novices may know what they like/prefer in abstract paintings, but do not generally feel that abstract paintings are beautiful. If so, then the quest to identify the dimensions of abstract art that lead to higher beauty ratings may be somewhat quixotic, at least among art novices. Regardless, these dissociations lead us to concur with others who have recommended that researchers collect more than one subjective rating outcome [16]. In our case, what people like in paintings, and what they find beautiful, was easier to identify for representational paintings than for abstract paintings.

Our study also raises the question of why some of our measures were informative about aesthetic ratings whereas others were not. Many dimensions of our results could be clarified and explored through further study, including dissociations across painting type (e.g., why was horizontal symmetry preferred for representational paintings but not for abstract paintings? why did participants like abstract paintings with lower brightness SD, but like representational paintings with higher brightness SD?). It could be that these variables manifest in different ways across the two types of images. For example, horizontal symmetry could signal a water scene for representational paintings (e.g., a forest reflected on a lake), but not for abstract paintings. As another example, the subjective ratings of meaningfulness could have been made on a different basis for abstract paintings (e.g., “how much does this painting resonate with me?”) than for representational paintings (e.g., “how well do I understand what’s depicted in this painting?”). In addition, one could explore dissociations across ratings for a given painting type (e.g., why did only edge density distinguish liking vs. beauty ratings for representational paintings?). It would also be informative to isolate and manipulate the influence of such factors on ratings via experiments, where possible, both to test the replicability of these more “micro findings”, as well as to enable causal inferences to be drawn about them. In turn, such findings could be used to shape and constrain accounts of aesthetic judgment [25, 37, 38, 39].

## Conclusion

Our study provides an important step up in the study of predictors of aesthetic judgments, by including both subjective and objective predictors, by collecting more than one subjective outcome measure, and by comparing ratings for distinct types of artworks. As noted above, our study has limitations such as the fact that our results necessarily depend on our choice of predictors and on our selection of paintings. We call on others to explore these and other aspects of our findings in more detail. Our approach could also be extended to explore other types of influences on aesthetic judgments. For example, predictors of beauty and/or liking may well differ for art experts than for the art novices that dominated our sample. As another example, presenting a mixture of abstract and representational artworks might lead participants to adopt a homogenous set of criteria for evaluating both types [18]. Thus, contextual influences on aesthetic judgments remain an important avenue for exploration [17].

## Supporting information

S1 Table Abstract paintings: Correlations between dependent variables, objective predictors, and subjective predictors. (DOCX)

S2 Table Representational paintings: Correlations between dependent variables, objective predictors, and subjective predictors. (DOCX)

S1 Appendix Example high and low painting for each significant predictor by painting type. (DOCX)

S2 Appendix LASSO regression analyses. (DOCX)
