# Supplementary material for: Prediction of beauty and liking ratings for abstract and representational paintings using subjective and objective measures
Source: PLoS One. 2018 Jul 6;13(7):e0200431. doi: 10.1371/journal.pone.0200431 (PMC6034882; doi:10.1371/journal.pone.0200431)
Supplement: S1 Table — (DOCX) [file pone.0200431.s001.docx]

**S1 Table. Abstract paintings: Correlations between dependent variables, objective predictors, and subjective predictors.**

| Measure | 1 | 2 | 3 | 4 | 5 | 6 | 7 | 8 | 9 | 10 | 11 | 12 | 13 | 14 | 15 |
| --- | --- | --- | --- | --- | --- | --- | --- | --- | --- | --- | --- | --- | --- | --- | --- |
| (1) Beauty | – |  |  |  |  |  |  |  |  |  |  |  |  |  |  |
| (2) Liking | .27*** | – |  |  |  |  |  |  |  |  |  |  |  |  |  |
| (3) Meaningfulness | .14* | .54*** | – |  |  |  |  |  |  |  |  |  |  |  |  |
| (4) Complexity | .30*** | .32*** | .55*** | – |  |  |  |  |  |  |  |  |  |  |  |
| (5) Emotionality | .35*** | .62*** | .68*** | .59*** | – |  |  |  |  |  |  |  |  |  |  |
| (6) Color Warmth | .04 | –.17** | .01 | .13 | –.13* | – |  |  |  |  |  |  |  |  |  |
| (7) Saturation Mean | .07 | –.03 | .08 | .08 | –.02 | .57*** | – |  |  |  |  |  |  |  |  |
| (8) Brightness Mean | –.04 | .10 | –.15* | –.28*** | –.18** | .21** | –.06 | – |  |  |  |  |  |  |  |
| (9) Hue SD | –.01 | .12 | .05 | .08 | –.00 | .02 | –.10 | –.12 | – |  |  |  |  |  |  |
| (10) Saturation SD | .05 | .05 | .08 | .14* | –.02 | .39*** | .54*** | –.08 | .21** | – |  |  |  |  |  |
| (11) Brightness SD | .01 | –.10 | .08 | .02 | .08 | –.00 | .12 | –.17** | .12 | .38*** | – |  |  |  |  |
| (12) RGB Component | .02 | .39*** | .12 | –.00 | .18** | –.73*** | –.32*** | –.03 | .11 | –.10 | –.09 | – |  |  |  |
| (13) Straight Edge Density | .16* | –.01 | .16* | .52*** | .12 | .19** | .12 | –.12 | –.01 | .24*** | .08 | –.13 | – |  |  |
| (14) Non-Straight Edge Density | .10 | .02 | –.11 | .09 | .08 | –.01 | –.10 | .07 | –.19** | –.35*** | –.41*** | .00 | –.25*** | – |  |
| (15) Vertical Symmetry | –.04 | .14* | –.12 | –.21** | –.08 | –.03 | –.26*** | .61*** | –.17* | –.45*** | –.57*** | .10 | –.25*** | .35*** | – |
| (16) Horizontal Symmetry | –.05 | .02 | –.28*** | –.21** | –.19** | –.04 | –.33*** | .64*** | –.21** | –.47*** | –.61*** | .05 | –.15* | .31*** | .84*** |

** p* < .05; ** *p* < .01; *** *p* < .001
