# Supplementary material for: Prediction of beauty and liking ratings for abstract and representational paintings using subjective and objective measures
Source: PLoS One. 2018 Jul 6;13(7):e0200431. doi: 10.1371/journal.pone.0200431 (PMC6034882; doi:10.1371/journal.pone.0200431)
Supplement: S2 Table — (DOCX) [file pone.0200431.s002.docx]

**S2 Table. Representational paintings: Correlations between dependent variables, objective predictors, and subjective predictors.**

| Measure | 1 | 2 | 3 | 4 | 5 | 6 | 7 | 8 | 9 | 10 | 11 | 12 | 13 | 14 | 15 |
| --- | --- | --- | --- | --- | --- | --- | --- | --- | --- | --- | --- | --- | --- | --- | --- |
| (1) Beauty | – |  |  |  |  |  |  |  |  |  |  |  |  |  |  |
| (2) Liking | .93*** | – |  |  |  |  |  |  |  |  |  |  |  |  |  |
| (3) Meaningfulness | .82*** | .79*** | – |  |  |  |  |  |  |  |  |  |  |  |  |
| (4) Complexity | .79*** | .71*** | .79*** | – |  |  |  |  |  |  |  |  |  |  |  |
| (5) Emotionality | .77*** | .73*** | .88*** | .75*** | – |  |  |  |  |  |  |  |  |  |  |
| (6) Color Warmth | .33*** | .37*** | .18** | .24*** | .11 | – |  |  |  |  |  |  |  |  |  |
| (7) Saturation Mean | .09 | .08 | .08 | .20** | .05 | .62*** | – |  |  |  |  |  |  |  |  |
| (8) Brightness Mean | –.00 | .05 | –.15* | –.22** | –.22** | .28*** | –.07 | – |  |  |  |  |  |  |  |
| (9) Hue SD | .02 | .06 | .02 | .01 | –.01 | –.16* | –.47*** | –.09 | – |  |  |  |  |  |  |
| (10) Saturation SD | .17* | .17** | .07 | .25*** | .00 | .50*** | .52*** | .12 | –.01 | – |  |  |  |  |  |
| (11) Brightness SD | .43*** | .43*** | .29*** | .36*** | .24*** | .32*** | .14* | .09 | .04 | .30*** | – |  |  |  |  |
| (12) RGB Component | .05 | .05 | –.01 | –.03 | –.05 | –.56*** | –.51*** | .03 | .42*** | –.16* | –.02 | – |  |  |  |
| (13) Straight Edge Density | .01 | .01 | .03 | .13* | –.10 | .14* | .23*** | .02 | .08 | .27*** | .05 | –.01 | – |  |  |
| (14) Non-Straight Edge Density | –.11 | –.24*** | –.18** | –.01 | –.13* | –.01 | .06 | .05 | –.08 | –.02 | –.06 | .00 | –.37*** | – |  |
| (15) Vertical Symmetry | –.15* | –.06 | –.23*** | –.38*** | –.18** | .00 | –.23*** | .59*** | –.10 | –.20** | –.21** | .04 | –.32*** | .03 | – |
| (16) Horizontal Symmetry | –.13 | –.07 | –.12 | –.32*** | –.14* | –.08 | –.30*** | .56*** | –.09 | –.34*** | –.50*** | .05 | –.22** | .09 | .68*** |

** p* < .05; ** *p* < .01; *** *p* < .001
