# Supplementary material for: Prediction of beauty and liking ratings for abstract and representational paintings using subjective and objective measures
Source: PLoS One. 2018 Jul 6;13(7):e0200431. doi: 10.1371/journal.pone.0200431 (PMC6034882; doi:10.1371/journal.pone.0200431)
Supplement: S1 Appendix — (DOCX) [file pone.0200431.s003.docx]

**S1 Appendix. Example high and low painting for each significant predictor by painting type.**

**Abstract Paintings**

|  | Low Example | High Example |
| --- | --- | --- |
| Meaningfulness (subjective) | [Robert Motherwall, 1953-1954, Elegy to the Spanish Republic No. 34](https://d32dm0rphc51dk.cloudfront.net/2ZRC-xyTYjKCfovUQOzoWg/larger.jpg)  Meaningfulness = 3.32  Beauty = 2.90  Liking = 3.57 | [Imogen Hellebore, 1926, False Hellebore](http://www.artnet.com/artists/imogen-cunningham/false-hellebore-8vHk8LHNZxJQFKjiTFPrqg2)  Meaningfulness = 6.26  Beauty = 4.90  Liking = 6.52 |
| Emotionality (subjective) | [Robert Motherwell, 1949, The Voyage](https://www.moma.org/collection/works/36685)  Emotionality = 3.02  Beauty = 3.08  Liking = 3.22 | [Carol Schiff, Shade of Blue](http://2.bp.blogspot.com/-Z4IQWwXGnAg/ViuPRPcA_qI/AAAAAAAAHYo/EqsLvrte0g8/s1600/AB%2BBkuepurpturq.jpg)  Emotionality = 5.78  Beauty = 4.85  Liking = 5.69 |
| Hue SD (objective) | [Hans Hoffman, 1961, Capriccio](https://www.sfmoma.org/artwork/94.542)  Hue SD = 0.22  Beauty = 4.18  Liking = 3.42 | [Filomena de Andrade Booth, Flight](https://www.dailypainters.com/paintings/256415/Flight-Original-Abstract-Painting-by-Texas-Contemporary-Artist-Filomena-de-Andrade-Booth/Filomena-Booth)  Hue SD = 2.32  Beauty = 4.53  Liking = 5.30 |
| Saturation SD (objective) | [Georg Herold, 1990, Untitled](https://www.sfmoma.org/artwork/90.252)  Saturation SD = 0.06  Beauty = 3.02  Liking = 4.22 | [Georgia O’Keefe, 1960, Blue, Black and Grey](https://www.google.com/culturalinstitute/beta/asset/blue-black-and-grey/1wHtxu2jZaZ0LA)  Saturation SD = 0.39  Beauty = 3.46  Liking = 5.96 |
| Mean Brightness (objective) | [Georg Herold, 1990, Untitled](https://www.sfmoma.org/artwork/90.252)  Mean Brightness = 0.28  Beauty = 3.02  Liking = 4.22 | [Filomena de Andrade Booth, Flight](https://www.dailypainters.com/paintings/256415/Flight-Original-Abstract-Painting-by-Texas-Contemporary-Artist-Filomena-de-Andrade-Booth/Filomena-Booth)  Mean Brightness = 0.85  Beauty = 4.53  Liking = 5.30 |
| Brightness SD (objective) | [Kurt Schwitters, 1919, Revolving](https://www.moma.org/collection/works/79211)  Brightness SD = 0.08  Beauty = 3.35  Liking = 5.00 | [Robert Motherwell, 1957-1961, Elegy to the Spanish Republic, No. 57](https://www.sfmoma.org/artwork/94.383)  Brightness SD = 0.38  Beauty = 3.79  Liking = 3.30 |
| RGB Component (objective) | [Paul Klee, 1937, Fragments](https://www.sfmoma.org/artwork/64.5)  RGB Component = -2.23  Beauty = 3.36  Liking = 3.98 | [Rinella Ivankovic, The Sounds](http://www.abstractartistgallery.org/wp-content/uploads/2013/02/Abstract-Art-Painting-Rinella-Ivankovic-The-Sounds.jpg)  RGB Component = 3.58  Beauty = 4.58  Liking = 5.98 |
| Entropy (objective) | [Robert Motherwell, 1953-1954, Elegy to the Spanish Republic No. 34](https://d32dm0rphc51dk.cloudfront.net/2ZRC-xyTYjKCfovUQOzoWg/larger.jpg)  Entropy = 5.35  Beauty = 2.90  Liking = 3.57 | [Lee Pina, Memory Pool](http://www.leepina.com/abstract/memory-pool)  Entropy = 7.87  Beauty = 6.72  Liking = 5.89 |

**Representational Paintings**

|  | Low Example | High Example |
| --- | --- | --- |
| Meaningfulness (subjective) | [Theodore Rousseau, 1837, The Board of Bellecroix](http://allpainters.org/wp-content/themes/paint/paintings/full/the-board-of-bellecroix-1848.jpg)  Meaningfulness = 3.31  Beauty = 4.06  Liking = 3.23 | [Andrew Melrose, 1867, Westward the Star of Empire Takes Its Way - Near Council Bluffs, Iowa](http://www.the-athenaeum.org/art/full.php?ID=39951)  Meaningfulness = 7.07  Beauty = 6.63  Liking = 6.07 |
| Complexity (subjective) | [Winston Churchill, Winter Sunshine, Chartwell](https://www.wikiart.org/en/winston-churchill/winter-sunshine-chartwell)  Complexity = 3.29  Beauty = 2.80  Liking = 3.11 | [Frederic Edwin Church, 1861, Oil Study of Cotopaxi](https://commons.wikimedia.org/wiki/File:Oil_Study_of_Cotopaxi_Frederic_Edwin_Church.jpg)  Complexity = 7.29  Beauty = 6.10  Liking = 5.68 |
| Emotionality (subjective) | [The Port of Morgat, 1882, Odilon Redon](https://en.wikipedia.org/wiki/Odilon_Redon#/media/File:16_sep_13_redon_morgat.jpg)  Emotionality = 3.73  Beauty = 4.16  Liking = 4.36 | [Joseph Wright of Derby, 1774-1776, Vesuvius from Portici](https://en.wikipedia.org/wiki/Vesuvius_in_Eruption_(Wright_painting)#/media/File:Joseph_Wright_of_Derby_-_Vesuvius_from_Portici.jpg)  Emotionality = 6.42  Beauty = 5.80  Liking = 5.70 |
| Color warmth (subjective) | [John Constable, 1835, Stonehenge](https://www.gettyimages.ca/detail/news-photo/john-constable-english-school-stonehenge-1835-watercolour-news-photo/624464310#john-constable-english-school-stonehenge-1835-watercolour-london-and-picture-id624464310)  Color warmth = 2.46  Beauty = 4.22  Liking = 3.55 | [Thomas Moran, Indian Village](http://www.wikigallery.org/wiki/painting_337233/Thomas-Moran/Indian-Village)  Color warmth = 7.72  Beauty = 7.00  Liking = 6.95 |
| Brightness SD  (objective) | [James Whistler, 1884, Wortley - Note in Green](http://www.museumsyndicate.com/item.php?item=69433)  Brightness SD = 0.16  Beauty = 3.29  Liking = 3.23 | [Thomas Doughty, 1834, Romantic Landscape with a Temple](http://www.mfa.org/collections/object/romantic-landscape-with-a-temple-33842)  Brightness SD = 0.33  Beauty = 5.94  Liking = 5.72 |
| RGB Component (objective) | [Joachim Patinir, 1520, Landscape with the Destruction of Sodom and Gomorrah](https://en.wikipedia.org/wiki/Joachim_Patinir#/media/File:Joachim_Patinir_010.jpg)  RGB Component = -2.15  Beauty = 4.18  Liking = 4.21 | [Paul Bril, 1601, A View on the Rhine](https://artuk.org/discover/artworks/view-on-the-rhine-5651)  RGB Component = 2.22  Beauty = 5.61  Liking = 5.28 |
| Straight Edge Density (objective) | [Joseph Wright of Derby, 1782, Arkwright’s Cotton Mills by Night](https://www.wikiart.org/en/joseph-wright/arkwright-s-cotton-mills-by-night)  Straight Edge Density = 0.01  Beauty = 4.63  Liking = 5.19 | [Giovanni Bellini, 1475-1480, St. Francis in Ecstasy](https://en.wikipedia.org/wiki/St._Francis_in_Ecstasy#/media/File:Giovanni_Bellini_-_Saint_Francis_in_the_Desert_-_Google_Art_Project.jpg)  Straight Edge Density = 0.08  Beauty = 4.80  Liking = 4.60 |
| Non-Straight Edge Density (objective) | [John Martin, 1810’s, Ruins of an Ancient City](https://commons.wikimedia.org/wiki/File:Ruins_of_an_Ancient_City_by_John_Martin,_1810s.JPG)  Non-Straight Edge Density = 0.04  Beauty = 6.02  Liking = 6.36 | [John Constable, 1835, Stonehenge](https://www.gettyimages.ca/detail/news-photo/john-constable-english-school-stonehenge-1835-watercolour-news-photo/624464310#john-constable-english-school-stonehenge-1835-watercolour-london-and-picture-id624464310)  Non-Straight Edge Density = 0.12  Beauty = 4.22  Liking = 3.55 |
| Horizontal Symmetry (objective) | [Joachim Patinir, 1520, Landscape with the Destruction of Sodom and Gomorrah](https://en.wikipedia.org/wiki/Joachim_Patinir#/media/File:Joachim_Patinir_010.jpg)  Horizontal Symmetry = 0.72  Beauty = 4.18  Liking = 4.21 | [Albrecht Dürer, 1495, View of the Arco Valley](https://commons.wikimedia.org/wiki/File:Burg_arco_d%C3%BCrer_1495.JPG)  Horizontal Symmetry = 0.94  Beauty = 5.88  Liking = 5.62 |
