# Supplementary material for: Prediction of beauty and liking ratings for abstract and representational paintings using subjective and objective measures
Source: PLoS One. 2018 Jul 6;13(7):e0200431. doi: 10.1371/journal.pone.0200431 (PMC6034882; doi:10.1371/journal.pone.0200431)
Supplement: S2 Appendix — (DOCX) [file pone.0200431.s004.docx]

**S2 Appendix. LASSO Regression Analyses.**

Least absolute shrinkage and selection operator (LASSO) [1] is a form of penalized regression [2]. It finds coefficients that minimize the sum of squared residuals while minimizing the sum of the absolute values of coefficients in the model. This latter sum is multiplied by a value (i.e., lambda), and this penalty in effect determines the extent to which coefficients are “shrunk”. The optimal value of lambda is determined via cross-validation, and a value is chosen according to a benchmark. Here we set that benchmark as: the largest value of lambda that results in a mean cross-validated error within 1 standard error of the minimum. We iterated this process 501 times and then chose the median optimal lambda value. With this penalty, some coefficients will be shrunk to zero; thus, LASSO also performs variables section. We used this approach to generate models for each combination of rating and painting type, using *R* (R Core Team, 2016) and the package *glmnet* [3].

**Table S1. LASSO regression models predicting beauty or liking, for abstract or representational paintings. Predictors with coefficients shrunk to 0.00 are not included.**

| Predictor | B |
| --- | --- |
| Abstract/Beauty | |
| **Emotion** | 0.28 |
| Abstract/Liking | |
| **Meaningfulness** | 0.17 |
| **Emotion** | 0.43 |
| **Brightness Mean** | 0.48 |
| **Hue SD** | 0.04 |
| **Brightness SD** | -0.14 |
| **RGB Component** | 0.13 |
| Vertical Symmetry | 0.13 |
| Representational/Beauty | |
| **Meaningfulness** | 0.39 |
| **Complexity** | 0.25 |
| **Emotion** | 0.24 |
| **Color** | 0.11 |
| Brightness Mean | 0.29 |
| **Brightness SD** | 1.54 |
| **RGB Component** | 0.14 |
| **Horizontal Symmetry** | 0.20 |
| Representational/Liking | |
| **Meaningfulness** | 0.46 |
| **Complexity** | 0.16 |
| **Emotion** | 0.26 |
| **Color** | 0.21 |
| Saturation Mean | -0.11 |
| **Brightness SD** | 2.19 |
| **RGB Component** | 0.22 |
| **Straight Edge Density** | -1.23 |
| **Non Straight Edge Density** | -4.18 |
| Vertical Symmetry | 0.85 |
| **Horizontal Symmetry** | 0.59 |

*Note*. Bold indicates significant predictors in the stepwise regression model in the main text.

**References**

1. Tibshirani, R. (1996). Regression shrinkage and selection via the lasso. *Journal of the Royal Statistical Society. Series B (Methodological)*, *58*, 267-288.

2. Friedman, J., Hastie, R., & Tibshirani, R. (2010). Regularization Paths for Generalized Linear Models via Coordinate Descent. *Journal of Statistical Software, 33*, 1-22. URL <http://www.jstatsoft.org/v33/i01/>.

3. Friedman, J., Hastie, T., & Tibshirani, R. (2010). Regularization Paths for Generalized Linear Models via Coordinate Descent. *Journal of Statistical Software, 33*, 1-22.
